# How can qualitative in-depth interviews optimize cross-cultural measurement of academic resilience?

**DOI:** 10.3389/fpsyg.2025.1444978

**Published:** 2025-03-20

**Authors:** Tian Fang Liu, Song Ching Fan, Xin Le Jiang

**Affiliations:** ^1^Department of Social Work, Social and Public Administration School, Ling Nan Normal University, Zhanjiang, China; ^2^Department of Human Resource Management, Social and Public Administration School, Ling Nan Normal University, Zhanjiang, China

**Keywords:** academic resilience, cross-cultural study, qualitative in-depth interviews, measurement scale, confirmatory factor analysis (CFA)

## Abstract

This study explores how qualitative in-depth interviews can optimize the measurement of academic resilience across cultures, addressing the challenges faced by Chinese university students in academic setbacks. Employing a mixed-methods approach, we conducted in-depth interviews analyzed through grounded theory using Nvivo 14 and developed a measurement scale for academic resilience among Chinese students. Confirmatory Factor Analysis (CFA) and Structural Equation Modeling (SEM) using IBM SPSS AMOS 28 were performed to validate the scale’s psychometric properties. The findings reveal that Chinese students exhibit unique cultural traits in coping with academic setbacks, such as face-saving concerns, guilt, self-blame, and a preference for stability. Their coping strategies involve self-reflection, cognitive restructuring, and external support seeking, shaped by cultural influences. The **Chinese University Students’ Academic Setback Resilience Scale (CUSASRS)** comprises dimensions of proactive coping strategies, learning engagement, and perceptions of academic adversity, offering a comprehensive representation of psychological and behavioral responses. This study contributes to understanding the cultural influences on academic resilience among Chinese students and provides empirical evidence for scale design and intervention strategies.

## 1 Introduction

This study aims to understand the experiences of frustration and coping among Chinese university students through qualitative in-depth interviews. Based on the coding of interview content and combined with Western scales, we designed a culturally appropriate academic resilience assessment tool for Chinese students. The “2023 Blue Book of China’s Mental Health (2023)” reports a 24% depression risk detection rate among individuals aged 18–24, highlighting the need for focused attention on this group. Similarly, in the United States, the highest depression prevalence is also in this age group, at 21.5% (Lee, 2023). This demographic often overlaps with university students, who are undergoing significant life transitions and identity formation (Robotham and Julian, 2006). Understanding their experiences is crucial for providing better support for their development. University students need to cultivate strong psychological qualities to handle future careers and life (Tight, 2020). Academic performance reflects students’ knowledge acquisition and overall development, predicting future career success (York et al., 2019).

Resilience is especially important for mental health research in young populations like college students (Tarriño-Concejero et al., 2024). Students with high academic resilience often achieve greater success and experience fewer psychological issues (Collie et al., 2017). Academic resilience is crucial for maintaining learning commitment amid setbacks (Hirvonen et al., 2020). It is also a key indicator of successful aging and future career success (Reich et al., 2020). Atkinson’s (1957) achievement motivation theory suggests that motivation is influenced by expectations of success and task value. Academic resilience, rooted in this theory, reflects a student’s capacity to sustain motivation and engagement in learning (Pitzer and Skinner, 2017). Rigorous research on college students is essential for identifying obstacles and developing support strategies to enhance success, retention, and well-being. High academic resilience helps students actively cope with difficulties, employ positive strategies, and reduce anxiety (Grayson, 2023). However, academic resilience in college students is often overlooked. It refers to the ability to overcome obstacles and negative experiences in learning (Connor and Davidson, 2003; Martin and Marsh, 2008; Cassidy, 2016; Shannon, 2020; Ahmed and Shawl, 2023; Latif and Amirullah, 2024). Given the unique demands of higher education, more research is needed on the relationship between resilience, student health, and perseverance towards academic goals (Shannon, 2020). College students with high self-efficacy are more likely to persevere through learning difficulties (Bandura, 2001).

Despite some studies indicating that resilience does not significantly impact academic stress (Fatimah et al., 2024), many scholars argue that high academic resilience helps individuals effectively cope with study-related difficulties (Yunanto et al., 2024; Carsone et al., 2024). Individuals with high self-efficacy typically maintain an optimistic view of their abilities and can better overcome obstacles. A positive mindset enhances personal resilience by promoting motivation and maintaining hope in challenging environments. Students with strong beliefs in their academic capabilities are more likely to persist in their efforts, particularly when facing obstacles (Shengyao et al., 2024). College students lacking self-regulation skills often feel helpless when facing learning setbacks, leading to lower resilience (Zimmerman, 2000). Self-regulated learning skills, such as monitoring the learning process, setting goals, and evaluating progress, increase resilience to setbacks (Schunk and DiBenedetto, 2020). In the realm of Chinese university students, traditional Chinese culture emphasizes self-improvement through diligent effort, fostering resilience. The cultural practice of ‘mutual assistance,’ where individuals share burdens and offer support, provides emotional sustenance during setbacks, bolstering resilience. These traditional values significantly contribute to individuals’ ability to navigate challenges. The “2023 Blue Book of China’s Mental Health” underscores the scarcity and varying quality of mental health services in China, emphasizing the need for tailored measurement tools to assess and enhance college students’ mental well-being. An exemplary tool is Cassidy’s (2016) 30-item Academic Resilience Scale (ARS-30), which assesses students’ adaptive cognitive, emotional, and behavioral responses to academic challenges (Matusevych et al., 2024).

However, Some scholars consider the Connor-Davidson Resilience Scale (CD-RISC) an effective tool for measuring resilience due to its strong structural validity, internal consistency, reliability, and cross-cultural validity (Tarriño-Concejero et al., 2024). Both the Academic Resilience Scale (ARS-30) and CD-RISC have not been adapted for different ethnic groups and cultural contexts. Shannon (2020) developed the College Student Resilience Scale (RSCS) based on CD-RISC to assess resilience levels in college students. In Western societies, higher education is typically seen as an individual pursuit, whereas in collectivist China, it is often aimed at enhancing social standing and providing financial support for the family (Guo et al., 2024). This cultural difference affects Chinese students’ perspectives on academic challenges. The Chinese social and cultural environment, influenced by traditional values, often leads to psychological issues manifesting as physical symptoms (Xu, 2024). There is a growing call for the indigenization of psychological theories to enhance their relevance and applicability in non-Western societies. This involves exploring local realities, perspectives, and epistemologies, thus broadening the discipline’s universality and ecological validity. Researchers must investigate the unique cultural processes shaping psychological phenomena within specific sociocultural systems. Grounding psychological research in indigenous philosophical traditions, value systems, and lived experiences is crucial for advancing understanding in Chinese societies (Zhao et al., 2023).

Existing resilience scales like ARS-30, CD-RISC, or RSCS primarily stem from Western contexts, potentially introducing cultural biases. Few studies have explored how race impacts resilience or how racial background influences resilience conceptualizations (Shannon, 2020). Future research should aim for racially and ethnically diverse samples to understand how resilience levels and academic success may vary across different racial groups. Yin et al. (2022) examined resilience development among Chinese university students, utilizing a scale and semi-structured interviews with 1,374 students and 20 individuals who experienced adversity, respectively. However, the study’s coding process lacked sophistication and did not adhere to standard systematic research practices. The interviews lacked depth, focusing mainly on individual events and coping strategies, neglecting thorough exploration of coping mechanisms and self-efficacy formation. Future studies should employ phenomenology to probe deeper into personal experiences, support systems, and practical coping strategies, using open-ended questions to facilitate comprehensive responses. Cultural nuances like emotional restraint and “saving face” were overlooked, essential for understanding resilience in this context. Additionally, resilience assessment should encompass everyday stressors and coping skills for comprehensive adaptation.

In order to advance psychology research and theoretical construction that truly align with the social and cultural reality of China, it is crucial to be rooted in the local philosophical traditions, value systems, and life experiences that differ from Western backgrounds. Therefore, in response to the national conditions of China and the unique group of university students, researching and developing a localized scale tool for academic resilience can more accurately assess and promote the psychological health of Chinese university students, enhancing their ability to cope with academic pressure and setbacks. In conclusion, redesigning an academic resilience scale that aligns with the characteristics of Chinese culture is a practical necessity in current psychological measurement research in China, and is crucial for better understanding and nurturing the academic qualities of Chinese university students.

This paper makes several notable contributions. By conducting qualitative interviews to delve into the subjective feelings and coping experiences of Chinese university students during academic setbacks, it addresses the limitations associated with relying solely on quantitative questionnaire surveys. The localized content derived from qualitative interviews offers insights closely aligned with the actual circumstances of Chinese university students, enriching the cultural relevance of the scale items. The integration of qualitative interviews with Western scale content demonstrates an effective approach to developing psychological measurement tools within a cross-cultural context. The development of a new scale provides a more robust measurement tool for examining the resilience of Chinese university students in academic setbacks, thereby enhancing the internal validity of the research, facilitating targeted intervention measures by educational institutions, and promoting the holistic development of students.

The remaining sections of this paper are arranged as follows: Part two introduces the relevant literature of this study. Part three reveals how phenomenological research conducts interview coding for new scale design. Part four delineates in detail the process through which the scale was developed. Part five presents the empirical results. Part six discusses the research findings. Part seven summarizes the research undertaken in this paper.

## 2 Literature review

### 2.1 The paradox of psychological measurement scales: balancing objectivity and subjectivity

Psychological scales objectively quantify individual traits, emotions, and behaviors, playing a crucial role in research, diagnosis, and treatment planning. However, inherent biases in self-report and limitations in representing psychological complexities pose challenges (Tafreshi, 2022; Jana and Aron, 2022). Despite systematic development processes, including construct definition and large-scale psychometric analysis, issues like item format and decontextualization persist, leading to response biases (Boyle et al., 2015; Davey, 2021; Jana and Aron, 2022; Morey et al., 2022; Mason et al., 2023). To address these limitations, integrating quantitative scales with qualitative methods like in-depth interviews is crucial (Ziegler and Hagemann, 2015). Mixed-methods research designs enrich our understanding, with clinical interviews providing complementary perspectives on quantitative scores (Morey et al., 2022) and enhancing personality trait assessment (DeYoung et al., 2022). Qualitative interviews uncover individual experiences missed by standardized scales and better identify respondents’ psychological states (Wright-Berryman et al., 2023). Combining scales with qualitative methods offers a more holistic understanding, underscoring the importance of mixed-methods approaches in psychological measurement research. Despite their susceptibility to distortion and bias, self-report scales remain indispensable in studying mental disorders due to their quantitative nature, facilitating sophisticated modeling and advancing our understanding of mental illness (Wright and Woods, 2020). While psychiatric diagnostic scales are invaluable, they have limitations; however, when integrated with other data sources, they are essential for advancing knowledge and guiding practice (Patrick, 2022). Despite their drawbacks, psychological scales remain the gold standard in adult personality assessment (DeYoung et al., 2022). Although standardized tests may sacrifice some ecological validity, their contribution to scientific progress is undeniable, necessitating validation against other data sources. Thus, scales remain vital in psychology and related fields, but their utilization alongside other data sources is essential to mitigate their limitations.

### 2.2 Enriching psychological inquiry: integrating quantitative and qualitative approaches

Quantitative questionnaires offer convenient data collection but have limitations in exploring subjective experiences, echoing Husserl’s emphasis on understanding phenomena through personal reflection (Fink, 2020). Complementing surveys with qualitative approaches is crucial, as they aim to interpret individuals’ subjective definitions of their lives. Relying solely on questionnaires may yield guarded responses, hindering a complete understanding. Interviews can address these shortcomings, providing a more contextualized understanding of experiences. Integrating both methods allows researchers to capture broader patterns and nuanced details. Qualitative methods, like in-depth interviews, contribute to a comprehensive understanding, particularly in phenomenological research that highlights marginalized voices. By engaging with lived experiences and meanings, qualitative approaches reveal nuances missed by quantitative measures, especially in studying historically marginalized populations.

The integration of qualitative and quantitative methods enriches our understanding of psychological processes, leading to a more comprehensive view. This synergistic approach enhances research depth, validity, and ecological relevance. Phenomenological insights inform the development of measurement tools that capture core layers of human experience (Friberg and Englander, 2019). By incorporating qualitative understanding, psychological scales better represent diverse subjective experiences, strengthening their ecological validity and clinical utility. This integration produces scales sensitive to the nuances of human experience, advancing our understanding and informing interventions (Sanmugam et al., 2022; Finlay, 2023). Capturing the richness of subjective experiences is crucial for tool development. In-depth phenomenological interviews provide detailed descriptions, revealing insights missed by quantitative methods alone (Neubauer et al., 2019). Incorporating qualitative understanding enhances the ecological validity of assessment instruments, ensuring they resonate with research participants’ subjective experiences.

In phenomenological research aiming to capture lived experiences firsthand, in-depth interviews remain paramount (Wassler and Kuteynikova, 2020). This inductive method is vital for uncovering the complexity often missed by standardized surveys (Fetters and Molina-Azorin, 2017; Roulston, 2018; Saldaña, 2021), enabling researchers to construct a rich understanding grounded in authentic experiences. These interviews unveil nuanced details and contextual qualities, providing insights into behavioral motivations. Developing measurement tools from lived experiences enhances cultural relevance and validity (Rallis and Rossman, 2012; Morgan, 2013), yielding precise findings that advance knowledge and inform interventions in psychological sciences.

### 2.3 Optimizing qualitative research: detailed coding and semi-structured interviews

In-depth interview studies often lack detailed descriptions of the coding and core theme generation process, which undermines transparency and credibility (Leavy, 2020). The absence of clear articulation of analytical steps compromises the replicability and reliability of research findings (Schreier, 2012; Silverman, 2016; Creswell and Poth, 2016). Trustworthiness and rigor suffer when data analysis procedures lack transparency, impacting qualitative research contributions (Schreier, 2012; Silverman, 2016; Creswell and Poth, 2016). Documenting and communicating analytical approaches are essential for enhancing transparency and credibility, vital for advancing the qualitative paradigm and integrating diverse methodologies in psychological research. Connecting coding to theory or literature is crucial for demonstrating how research findings contribute to the broader field (Creswell and Báez, 2020). Comparing data to theoretical concepts helps reveal hidden themes and meanings, enriching qualitative analysis. Documenting the coding process and its theoretical foundation is essential for readers to grasp the basis of research findings (Saldaña, 2021). Therefore, researchers must explain the process and details of these analytical steps clearly in their reporting, including comprehensive documentation and explanation of coding, theme generation, and their theoretical underpinnings in in-depth interview studies. This ensures the credibility, reliability, and comprehensibility of research results. Transparent accounts of analytical procedures enhance the trustworthiness of qualitative findings, demonstrating how themes and interpretations are grounded in data, crucial for advancing the qualitative paradigm and integrating diverse methodologies in psychological research.

While some limitations exist, scholars emphasize the importance of semi-structured interview protocols in qualitative research, particularly in phenomenological studies. These protocols offer advantages over completely open-ended interviews, with semi-structured, one-on-one interviews being preferred for collecting phenomenological data (Larkin et al., 2021). Coding plays a crucial role in theoretical analysis, aligning data with theoretical concepts and uncovering underlying themes and meanings. Documenting the coding process along with its theoretical framework is vital for readers to grasp the foundation of research findings (Saldaña, 2021). Researchers must comprehensively elucidate these analytical steps in their reports, detailing the coding process, theme generation, and theoretical basis in-depth interview studies. Transparent documentation of these procedures ensures the credibility, reliability, and comprehensibility of research results, enhancing trust in qualitative findings and illustrating how themes and interpretations are rooted in the data.

### 2.4 Reconstructing resilience: developing a tailored scale for university students

**Figure 1:**
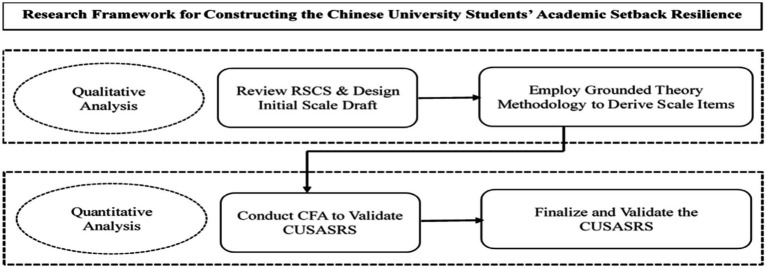
Research framework for CUSASRS.

Based on the reasons outlined above, this study aims to explore the frustrating scenarios and subjective experiences faced by Chinese university students in their learning through qualitative interviews with appropriate participants. The goal is to redesign scales that are more aligned with Chinese cultural characteristics, thereby gaining a deeper understanding of students’ authentic feelings, thoughts, and behavioral responses when facing and coping with frustrations. The qualitative data will be analyzed to identify themes, patterns, and trends, which will inform the development of items for the questionnaire scale. Figure 1 illustrates the framework for constructing the Academic Frustration Resilience Scale for Chinese University Students. Drawing on qualitative analysis and informed by Shannon (2020) Resilience to Stress in College Students (RSCS) scale, we will initiate the questionnaire scale. Grounded theory will inform the generation of scale items, integrating elements from existing scales to create a novel assessment tool. Subsequent quantitative analysis will assess the reliability and validity of the new scale, ultimately yielding an instrument tailored to assess the learning experiences of this demographic. This mixed-methods approach, combining qualitative exploration and quantitative validation, will facilitate a comprehensive understanding of the nuanced experiences and adaptive strategies of Chinese university students amidst academic frustrations. The resulting scale will serve as a valuable tool to support the learning and development of this population.

## 3 Scale design

### 3.1 Overlooking the impact of online surveys on the number of scale items

In recent years, online surveys have surged in popularity as a key method for questionnaire administration (Revilla and Höhne, 2020). However, the issue of questionnaire length and item quantity has often been overlooked, leading to potential problems such as respondent “speeding” or “satisficing,” where respondents spend less time on each question as the survey length increases. This trend can result in higher non-response rates, impacting data quantity and reliability. To address this, scholars have provided theoretical guidance on the optimal number of items in questionnaire design (Sharma, 2022). Recent research suggests that for online surveys, the ideal number of items falls between 10 and 16, and the optimal survey length ranges from 10 to 15 min (Galesic and Bosnjak, 2009; Hoerger, 2010; Rolstad et al., 2011; Revilla and Höhne, 2020). Neglecting to consider item quantity in online surveys can compromise data quality, leading to increased satisficing, non-response rates, and less reliable findings. Therefore, researchers should carefully balance the need for comprehensive measurement with the constraints of online survey environments to design questionnaires that are both informative and respondent-friendly.

The choice to reference the College Students’ Resilience Scale (RSCS) in this study’s scale design stems from its origin in the Connor-Davidson Resilience Scale (CD-RISC), a widely recognized tool for assessing resilience to stress and adversity. The CD-RISC’s application suggests its relevance for evaluating resilience to frustration. Specifically tailored for college students, the RSCS aims to measure resilience levels amidst adversity. While both the RSCS and CD-RISC assess resilience to frustration, the former caters explicitly to university students, unlike the more general CD-RISC. Thus, this study relies on the RSCS as a foundational tool for scale development, ensuring a tailored assessment of academic frustration resilience within the Chinese university student population. Leveraging the RSCS as a reference enables a comprehensive evaluation of academic frustration resilience among Chinese university students. By building upon established measures like the RSCS, this research enhances the relevance and validity of the new scale for its target population. Ensuring the cultural relevance of the scale entails meticulous item selection, aiming for alignment with the actual circumstances and values of Chinese students while eliminating potentially divergent items. For instance, items 16–18 in the RSCS, such as “Making my parents proud is important to me” and “My parents made sacrifices so I could pursue my dreams,” underscore parental influence, typical in Western contexts. However, in traditional Chinese culture, parental authority and filial piety are paramount, emphasizing obedience over personal aspirations. Directly importing such items may introduce cultural biases. Thus, careful adaptation is essential to ensure the scale resonates with Chinese university students’ experiences. Blindly adopting Western-derived items risks compromising cultural relevance and ecological validity, necessitating thoughtful adjustment and validation within the Chinese context.

Moreover, items 27 and 28 in the RSCS scale focus on the role of religious faith in resilience, such as “Believing in something larger than myself motivates me” and “My faith helps me when I am stressed.” However, in traditional East Asian circles, including China, religious belief may not serve as a primary source of resilience for most students. Chinese students often draw strength from Confucian culture, emphasizing personal moral cultivation and perseverance. Therefore, item design should prioritize aspects like self-regulation abilities and optimistic attitudes over religious content. To ensure cultural relevance and specificity, we meticulously considered Chinese students’ cultural characteristics, selecting appropriate item content while excluding potentially bias-inducing material. This rigorous process aims to create a scale that authentically mirrors the experiences of Chinese university students, bolstering its validity and utility within the local context. To maintain a high response rate and data quality, the preliminary questionnaire design will feature 15 items or fewer, as depicted in Table 1.

**Table 1:** Review RSCS and design initial scale draft.

Subject construct	Construct items
Unfavorable intra-individual factors	1. I frequently encounter adversity and difficulties in my academic life.
	2. Difficult academic tasks make me feel frustrated.
	3. I often feel discouraged by academic tasks.
Intrapersonal buoyancy factors	4. I will not give up my goals just because of temporary setbacks.
	5. I am capable of overcoming academic obstacles.
	6. Even when facing academic setbacks, I can quickly regain my determination.
	7. I always believe that I can emerge from academic lows.
Classroom buoyancy factors	8. My teachers always provide assistance when I encounter academic obstacles.
	9. My classmates encourage me not to give up on my studies.
	10. My family always supports my learning.
	11. My friends show concern for and encourage my academic performance.
Hedonic buoyancy factors	12. No matter what kind of academic setbacks I experience, I can still enjoy the pleasure of learning.
	13. The joy brought by learning often makes me forget the difficulties ahead.
	14. Even when encountering academic obstacles, I can continue to experience the joy of learning.
	15. Regardless of the future, I look forward to achieving good academic results.

### 3.2 Questionnaire design

The interview questions are designed to employ a phenomenological research approach, aiming to deeply explore the subjective experiential world, internal psychological processes, coping strategies, and growth trajectories of Chinese university students facing academic frustrations. This qualitative method seeks to reveal unique individual experiences and distinguish them from Western cultural contexts. Phenomenology emphasizes individuals’ subjective perceptions and meaning-making processes, aligning with Chinese cultural traditions. The 10 questions investigating university students’ internal experiences of academic frustration are grounded in theoretical foundations (Table 2). A phenomenological orientation facilitates an in-depth exploration of the nuanced, contextual nature of Chinese students’ lived experiences with academic challenges. Prioritizing participants’ perspectives can uncover cultural insights differing from Western-based understandings. The interview protocol, developed from these theoretical underpinnings, will gather rich, contextualized data on participants’ subjective experiences and adaptive strategies, laying the foundation for subsequent scale development.

**Table 2:** Theoretical basis and design rationale of the semi-structured questionnaire with 10 items.

Interview topic	Theoretical basis
1. Please describe a significant academic setback you recently encountered and how you felt at the time.	“The first question prompted participants to sketch a concrete failure experience and to express the feelings associated with that experience, so as to lay the groundwork for an in-depth exploration later on” (Bohlmeijer et al., 2008).
2. What struggles and confusion did you experience internally when facing this setback?	“Phenomenology is a study of people’s subjective experiences and interpretations of the world. That is, the study of phenomena” (Englander, 2020).
3. What impact did this academic setback have on your daily life, emotions, and self-worth?	Academic setbacks can tax students’ abilities to regulate their behavior, emotions, and cognitive resources and can negatively impact their self-concept (Yeager and Dweck, 2012).
4. What strategies or actions did you take to cope with this setback? What were the outcomes?	Understanding how people cope with stress is important for determining the effects of coping on adjustment and well-being” (Skinner et al., 2003).
5. What insights or revelations did you gain from overcoming this setback?	Phenomenological research involves exploring lived experience and its meanings. A central aim is to gain insightful descriptions of the way lived experience presents itself, by attending to perceptions and meanings that awaken our conscious awareness (Finlay, 2012).
6. Looking back on the entire experience, what do you feel was the biggest challenge? How did you overcome it?	Allowing participants to assess the peak challenges of their overall experience and articulate their coping mechanisms aids in presenting their subjective dilemmas and courses of action (Bandura, 2001)
7. Reflecting on the entire experience, what do you feel was the biggest challenge? How did you overcome it?	“The theoretical perspective guiding this review is that social support is a meta-construct referring to the process by which social relationships might promote health and well-being” (Cohen and Wills, 1985)
8. What inspirations have you drawn from this academic setback experience for your future learning and life? Are there any areas you have changed or re-evaluated?	The phenomenological approach allows the researcher to share the experiences under exploration in order to understand the essential truth of their meaning within the subjective perspective of those who have lived them (Jasper, 1994)
9. Based on your experience, what kind of help and support do you think college students need the most when facing academic setbacks?	Their stories and lived experiences can inform us about how we might best support them, what approaches work well, and where we might need to reconsider our practice (Lindseth and Norberg, 2004).
10. Is there anything else you would like to add or share about your experience with academic setbacks?	“Phenomenology aims to describe experiences as lived, in their fullest breadth and depth, attending carefully to perspectives of those who had the experiences” (Englander, 2020)

In summary, phenomenology emphasizes individuals’ subjective interpretation of experiences. Questions 1, 2, 3, 5, and 8 aim to capture participants’ authentic feelings, reflections, and transformative insights from frustration events - their subjective experiences. Questions 4 and 6 explore coping strategies and resilience, while Question 7 focuses on the influence of social support. Question 9 elicits recommendations for support channels, and Question 10 allows participants to share freely, enriching the data. Designing questions based on phenomenology provides deep insights into Chinese university students’ experiences with academic frustrations. This qualitative data will inform subsequent scale development.

### 3.3 Interview participants

Conducting in-depth case studies within specific contextual environments can reveal nuances overlooked in large-scale surveys, aiding theory building and intervention development (Duff, et al., 2022). This study will conduct two 30–40 min case interviews with male and female junior-level university students on March 18–19, 2024. Interviewers will create a non-judgmental environment conducive to detailed sharing (Seidman, 2006). Conducting interviews in March ensures students’ relative freedom from exam pressures, fostering favorable conditions for in-depth discussions. This time frame allows for concentrated attention and comprehensive accounts (Woodside, 2016; Roulston, 2018; Matza et al., 2023). Purposeful selection of junior-level students provides rich, contextualized data for scale development. The 30–40 min duration balances participants’ attention span with data depth required for quality interviews. It aligns with Moustakas’ (1994) emphasis on detailed descriptions of conscious experiences (Lin et al., 2013). Selecting junior-level students reflects their accumulated university experiences and nuanced coping strategies compared to lower-level undergraduates. This depth is crucial for exploring subjective academic frustrations. These considerations enable gathering rich, contextualized data vital for informing scale development.

### 3.4 Interview content coding

This study adopts grounded theory as the analytical framework. First, interview recordings will be transcribed verbatim and carefully reviewed multiple times to gain a comprehensive understanding. Important statements relevant to the research topic will be identified to reveal the essence of participants’ experiences. Commonly occurring keywords will be extracted to form the first level of open coding. These open codes will be gradually integrated into axial codes, which will be systematically categorized to establish broader core themes. Concise codes will summarize the primary meaning of each important statement, constructing the third level of selective coding. Frequency analyses on similar open codes will help organize them into potential themes to refine the scale.

Grounded theory offers a systematic method for extracting, categorizing, and synthesizing key elements from qualitative interview data. This inductive approach facilitates the development of a scale rooted in the experiences of Chinese university students, ensuring cultural relevance and ecological validity. Through rigorous analysis using open, axial, and selective coding, researchers can uncover prominent themes and patterns in academic frustration experiences. This qualitative groundwork informs the construction of a culturally-appropriate assessment tool.

In this study, NVivo 14 was used to code the interview content. The transcripts contained 81 statements, from which 53 key statements were identified. From these, 10 word frequencies were extracted, forming the initial open coding. These 10 open codes were consolidated into 5 axial codes, as detailed in Table 3. Using NVivo 14 enables a systematic approach to coding. Identifying important statements, extracting common keywords, and organizing them into progressively higher-level codes allows researchers to derive key themes and patterns systematically. The open coding stage captures initial concepts and ideas expressed by interviewees. Consolidating these into axial codes identifies broader, interconnected categories representing core aspects of academic frustration, as shown in Table 3. This iterative process ensures emergent themes and constructs are rooted in the experiences of Chinese university students, serving as a crucial basis for scale development.

**Table 3:** Interview content is coded for grounded theory development.

Axial coding	Open coding: word frequency	Open coding: key statements
Academic Setback Experience	1.Disappointment (15)	1.1 University Entrance Exam Failure/Mismatched Major Aspirations
	2. Low Mood (13)	2.1 Feelings of Loss/Disbelief/Frustration 2.2 Fear/Apprehension/Anxiety 2.3 Resentment/Confusion/Helplessness 2.4 Tardiness, Low Mood, Insomnia 2.5 Overcoming Psychological Disparities/Negative Challenges
	3. Failed Major Transition (9)	3.1 Transfer Policy Restrictions / Transfer Frustration
Internal Positive Forces	4. Self-Doubt (8)	4.1 Policy Ambiguity / Equity Inquiry 4.2 Identity Confusion / Self-Doubt 4.3 Understanding of Personal Characteristics 4.4 Reflection on Personal Motivations 4.5 Reassessment of Personal Values
	5. Abandonment of Expectations (4)	5.1 Contradictions Regarding University Retake 5.2 Loss of Learning Enthusiasm/Motivation
External Support	6. Lack of Support (6)	6.1 Seeking External Support (Family, Friends, Teachers) 6.2 Encouragement/Comfort/Support from Family 6.3 Sharing Experiences with Friends 6.4 Guidance/Advice from Teachers 6.5 Professional Psychological Counseling 6.6 Understanding and Support from Family 6.7 Comfort and Encouragement from Friends
Positive Emotional Experiences	7. Escaping Reality (5)	7.1 Contemplation of Taking a Leave of Absence 7.2 Challenges of Returning to Normal Life/Study
Proactive Behavioral Strategies	8. Inadequate Planning (7)	8.1 Ambiguity Regarding Future Development 8.2 Awareness of the Need for Resilience/Mental Toughness 8.3 Importance of Learning Planning 8.4 Enhancing Psychological Resilience/Ability to Cope with Setbacks 8.5 Acting Prudently/Understanding the Completeness of Information 8.6 Academic Development Guidance
	9. Blind Comparison (4)	9.1 Multidimensional Self-Realization
	10. Lack of Coping Strategies (3)	10.1 Rational Analysis of Reasons/Problems 10.2 Emotional Regulation (e.g., reading, engaging in hobbies) 10.3 Contingency Planning (e.g., considering graduate school) 10.4 Currently No Effective Means of Getting Through Completely

### 3.5 Selective coding: connotations of core themes

Converting the axial coding into selective coding aims to identify Chinese cultural characteristics from the interview content. These selective codes focus on cultural values, social norms, and behavioral patterns, providing deeper insights into how Chinese culture impacts individual cases. University students face various academic setbacks, leading to intense emotional pain and negative emotions like loss and anxiety. These emotions affect their daily state and future motivation. Some students adopt passive strategies, while others actively cope by seeking help and cultivating interests, regaining confidence and motivation. Students express a desire for external support, especially from family, friends, and professionals, when facing setbacks. These experiences prompt valuable growth and reflections, leading to adjustments in learning strategies and values for future development. Properly addressing setbacks promotes positive growth and development among university students, reflecting Chinese cultural characteristics.

#### 3.5.1 Cultural interpretation of painful emotional experiences

The open coding categories of “Disappointment,” including 1.1 University Entrance Exam Failure /Mismatched Major Aspirations, “Failed major transition,” including 3.1 Transfer Policy Restrictions /Transfer Frustration, and “Low mood,” including 2.1 Feelings of Loss /Disbelief /Frustration, 2.2 Fear/Apprehension/Anxiety, and 2.3 Resentment/Confusion/Helplessness, all reflect the painful emotional experiences of the university students. This can be explained by the emphasis on “self-restraint” in traditional Chinese culture, such as the Confucian concept of “Ke-Ji Fu-Li” (restraining oneself and returning to propriety), which emphasizes the need to regulate and cultivate oneself through self-cultivation in order to achieve moral rectitude. This also echoes the ingrained mindset in Chinese culture, where parents hope their children can suppress and control their emotions in order to promote social harmony (Yang et al., 2020). This notion of restraining one’s personal emotions has led Chinese university students to more easily experience intense feelings of guilt and self-blame when facing setbacks. The cultural imperative to control and restrict one’s emotions, rather than openly expressing them, contributes to the strong negative emotions experienced by these students in the face of academic difficulties and disappointments.

#### 3.5.2 Cultural roots of risk-aversion and pursuit of stability

The open coding categories of “Abandonment of expectations,” including 5.1 Contradictions Regarding University Retake and 7.1 Contemplation of Taking a Leave of Absence, reflect that some students tend to adopt passive strategies, such as avoiding reality, risk-aversion, and giving up on their expectations, failing to address the root problems. This can be attributed to the Chinese people’s clear “stability orientation,” where the pursuit of stability and harmony is one of the core Chinese cultural values. When addressing problems, there is a tendency to prioritize restoring order and harmony over driving change. This trait aligns with the Chinese cultural emphasis on “stability.” Valuing stability and fearing change, Chinese individuals often focus on maintaining order rather than fostering transformation when faced with challenges. This cultural orientation towards stability and risk aversion is evident in the coping strategies of university students in the study, who tend to avoid confronting core issues and adopt passive approaches to maintain stability.

#### 3.5.3 The need for help and the concept of “face”

The open coding categories of “Lack of support,” including 6.1 Seeking External Support (Family, Friends, Teachers), 6.2 Encouragement/Comfort/Support from Family, 6.3 Sharing Experiences with Friends, and 6.4 Guidance/Advice from Teachers, indicate that university students’ need for external help when facing setbacks may be influenced by the traditional Chinese concept of “face.” The notion of “face” is deeply rooted in Chinese culture, which makes many Chinese people reluctant to openly admit their own failures or shortcomings in public. This may also lead university students to not immediately seek help from others when facing setbacks, as they consider the issue of “face.” It is often not until the pressure of the unresolved problem becomes overwhelming that the individuals are forced to seek external resources for assistance (Hu, 1944; Hwang, 1987). The desire to maintain “face” and avoid publicly acknowledging difficulties can hinder Chinese university students from seeking needed support. This cultural dynamic can delay access to valuable resources and impair their ability to cope with academic setbacks effectively.

#### 3.5.4 Educational reflection and traditional Chinese educational ideologies

The open coding categories of “Self-doubt,” including 4.2 Identity Confusion/Self-Doubt, 4.3 Understanding of Personal Characteristics, 4.4 Reflection on Personal Motivations, and 4.5 Reassessment of Personal Values, as well as “Inadequate planning,” including 8.2 Awareness of the Need for Resilience/Mental Toughness, 8.3 Importance of Learning Planning, 8.4 Enhancing Psychological Resilience/Ability to Cope with Setbacks, and 8.5 Acting Prudently/Understanding the Completeness of Information, express that experiencing academic setbacks has also brought students valuable growth experiences and reflections. Experiencing setbacks prompts students to re-evaluate their learning strategies and educational values, leading to a renewed understanding of educational concepts. The intrinsic motivation for this reevaluation may stem from the traditional Chinese educational philosophy of “zi qiang bu. xi, zhi yu zhi shan” (self-strengthening and striving for perfection). “Zi qiang bu. xi” denotes a conscious effort to improve oneself, while “zhi yu zhi shan” embodies the pursuit of continuous perfection. In summary, the unique responses and internal experiences of Chinese university students facing academic setbacks are closely tied to traditional Chinese cultural values, including emotional restraint, a focus on stability and harmony, the importance of “face,” and the commitment to self-improvement. These cultural traits significantly shape their resilience in adversity.

### 3.6 New questionnaire content

First, this study has converted the coded themes and coded terms into questionnaire item content that aligns with the definition of academic resilience. Secondly, the coded items have been merged with the existing questionnaire items, and cross-compared, with repetitive or similar items being removed. The same items have been retained, and those that were too abstract, idealized, or deviated from the core essence of assessing academic setback tolerance have been deleted. Based on the core thematic content findings from the phenomenological study, 4 new items have been added, as detailed in Table 4. “When faced with setbacks, I will first comprehensively analyze the crux of the problem.” This new item considers the influence of the traditional Chinese “self-restraint” cultural concept. Chinese university students tend to restrain their personal emotions when facing setbacks, and rationally analyze the root causes of the problem, rather than being overwhelmed by emotions. This cultural influence from family education is deeply rooted in the Confucian idea of “self-restraint and return to propriety (ke-ji fu-li).” This theme corresponds to the 10.1 Rational Analysis of Reasons/Problems under the 5th axial code Proactive behavioral strategies in the interview coding.

**Table 4:** Generation of defined titles from open-ended coding.

**Axial coding**	**Reflecting the transformation of open coding into defined categories**
Academic Setback Experience	1.1 The challenge I face is how to regain the optimal state of life and learning. 1.2 My challenge lies in how to actively face setbacks and reshape a positive self-image. 1.3 The challenge I face is how to regain the optimal state of life and learning.
Internal Positive Forces	2.1 When facing academic challenges, I am able to confront them actively and persevere in my goals. 2.2 I have high expectations for my potential and abilities, believing that I can overcome obstacles. 2.3 I can harness inner motivation to reignite my passion for learning. 2.4 When confronted with setbacks, I weigh the pros and cons comprehensively to make the best choice. 2.5 Even in the face of temporary setbacks, I am confident that I can ultimately achieve my academic aspirations.
External Support	3.1 The careful guidance of my teachers has helped me find the right direction. 3.2 The sharing and inspiration from friends help me maintain a positive attitude. 3.3 The love and support of my family are the greatest motivation for overcoming difficulties.
Positive Emotional Experiences	4.1 No matter what learning setbacks I experience, I can still enjoy the pleasure of learning. 4.2 The joy brought by learning often makes me forget about the difficulties ahead. 4.3 Even when encountering learning obstacles, I can still continue to experience the joy of learning.
Proactive Behavioral Strategies	5.1 When facing setbacks, I first conduct a comprehensive analysis of the root of the problem. 5.2 When encountering setbacks, I actively seek external resources and advice to accumulate problem-solving experience. 5.3 I regulate my emotions through healthy and positive means to maintain a good mindset. 5.4 When facing developmental obstacles, I promptly seek favorable alternative paths for development.

Overall, the study systematically translated the core thematic connotations regarding university students’ academic setbacks from the phenomenological research findings into questionnaire items that reflect traditional Chinese cultural concepts such as “self-restraint,” “stability,” “face,” and “pursuit of perfection.” This was done to quantitatively measure students’ positive coping abilities in cognitive, emotional, and behavioral domains, in order to more accurately assess the manifestation of cultural characteristics in Chinese university students’ experiences of academic setbacks. This grounding in a local cultural perspective has helped to objectively and comprehensively interpret the inherent experiences of Chinese university students in their process of coping with academic setbacks. Consequently, the scale has further improved the assessment of university students’ inner qualities, emotional regulation, and proactive coping behaviors when facing setbacks in their academic endeavors.

## 4 Methodology

Confirmatory factor analysis (CFA) and structural equation modeling (SEM) are fundamental statistical methods in assessing the reliability and validity of psychological scales. They are widely recognized as the gold standard for evaluating scale dimensionality, reliability, validity, and testing hypothesized relationships among theoretical constructs, especially for multi-item instruments measuring latent constructs (Hoyle, 2012; Thakkar, 2020; Sarstedt and Ringle, 2020; Jobst et al., 2023; Kline, 2023). Factor analysis allows researchers to assess the construct validity of measurement instruments by examining how well the observed variables load onto the intended latent factors. Structural equation modeling further enables the testing of complex theoretical models involving multiple latent variables and hypothesized causal relationships (Kline, 2023). “Confirmatory factor analysis (CFA) is pivotal for assessing scale factorial structure and psychometric properties. When combined with structural equation modeling (SEM), it integrates measurement models and tests substantive hypotheses. Despite emerging computational methods, SEM remains the benchmark for validating evidence of validity and construct validity in psychological measurement, uniquely capable of evaluating both measurement and theoretical models simultaneously.

This research adopts a phenomenological perspective, using semi-structured interviews to explore adversity among Chinese university students. Qualitative interviews accurately capture academic setback experiences. By integrating these elements with Western scales, a culturally-adapted academic setback resilience scale is developed. Empirical testing confirms scale dimensions, reliability, and validity through factor analysis and SEM, verifying hypothesized relationships between constructs. Grounded in phenomenology, this approach deeply investigates Chinese university students’ experiences of academic adversity. Integrating qualitative and quantitative methods, along with culturally-sensitive scale development and rigorous statistical analysis, comprehensively examines academic setback resilience within the Chinese cultural context. Confirmatory factor analysis and structural equation modeling validate the scale’s psychometric properties and test the theoretical model, ensuring robustness and generalizability of findings.

## 5 Data

In March 2024, a pilot survey was conducted using an online questionnaire to collect data. Prior to the formal survey, 30 pre-test questionnaires were distributed. Factor analysis revealed a high level of internal consistency with a Cronbach’s *α* of 0.904, indicating reliability. For construct validity, the Kaiser-Meyer-Olkin (KMO) measure was 0.635, and Bartlett’s test of sphericity had a significant *p*-value (<0.05), suggesting adequate validity. However, Exploratory Factor Analysis (EFA) on the pre-test questionnaire was conducted to identify variables for inclusion in the new scale. Item 15, “When facing setbacks, I first conduct a comprehensive analysis of the root of the problem,” was removed due to insignificant factor loadings. Similarly, item 16, “When encountering setbacks, I actively seek external resources and advice to accumulate problem-solving experience,” showed significant correlations with multiple factors, indicating potential design flaws, and was also removed to maintain scale quality (Hair et al., 2006). Interpretations of items 15 and 16 were informed by in-depth interview content and thematic implications. Item 15’s low factor loadings may be linked to traditional Chinese cultural norms emphasizing the expression of intense negative emotions over rational problem analysis during setbacks. Item 16’s responses may reflect concerns about maintaining “face” in Chinese culture, influencing respondents’ reluctance to seek external help publicly. These cultural influences may affect respondents’ perceptions of items 15 and 16, impacting their performance in factor analysis.

## 6 Empirical results

In April, the students targeted for this study were invited to participate on a voluntary basis. All respondents provided informed consent prior to participating in the research. Regarding sample size and validity, a total of 545 questionnaires were collected, of which 520 were valid, and 25 invalid questionnaires were excluded, resulting in an effective sample size of 520. The sample included 214 males (41.15%) and 306 females (58.85%), showing a relatively balanced gender distribution. The sample distribution by academic year was as follows: 199 freshmen (38.26%), 85 sophomores (16.34%), 102 juniors (19.61%), and 134 seniors (25.79%).

Regarding the geographical location of the respondents’ families, 269 (51.73%) were from urban areas, and 251 (48.27%) were from rural areas, indicating a roughly equal urban–rural distribution. In terms of academic disciplines, 233 students (44.8%) were from the social sciences, 201 (38.65%) from the natural sciences, and 61 (11.73%) from the arts (including music, sports, and fine arts).

Regarding the unique demographic characteristic of China’s one-child policy, 103 respondents (19.8%) were only children, while 417 (80.2%) were not, indicating a majority of non-only children. The survey sample demonstrated a certain degree of diversity in major demographic characteristics such as gender, academic year, geographical location of the family, and academic discipline distribution. However, there was a significant skew in the distribution of the only-child characteristic.

The Kaiser-Meyer-Olkin (KMO) value was 0.896, and Bartlett’s test of sphericity yielded a chi-square value of 3384.214 with 91 degrees of freedom, with a significance level of *p* < 0.001, indicating that the sample was suitable for conducting Confirmatory Factor Analysis (CFA). The factor loadings for each item ranged from 0.564 to 0.830, generally meeting the ideal requirement of being above 0.5. The three factors corresponded well with the hypothesized theoretical model, demonstrating good construct validity. The Cronbach’s *α* coefficient for the CFA was 0.88, indicating that the scale had high internal consistency and could reliably measure students’ academic frustration tolerance. This also highlights the necessity of appropriately adjusting the scale’s content to reflect the specificity of regional cultural contexts. This necessity aligns with Husserl’s emphasis on “returning to the things themselves,” advocating for understanding the essence of phenomena through reflection and examination of personal experiences. This study’s use of qualitative interviews to extract the subjective experiences of Chinese university students regarding academic frustration supports the need for such adjustments to the scale.

Composite Reliability (CR) reflects the degree of internal consistency of latent variables, while Average Variance Extracted (AVE) reflects the extent to which a latent variable can explain the average variance of its measurement indicators. It is an indicator of convergent validity. According to the recommendations of Fornell and Larcker (1981), the composite reliability (CR) should be above 0.6, and the average variance extracted (AVE) should exceed 0.5. Generally, a CR greater than 0.7 indicates good internal consistency of the latent variables, suggesting that the scale has high reliability. An AVE greater than or equal to 0.5 indicates that the latent variable can explain more than 50% of the variance of its measurement indicators, suggesting that the scale has good convergent validity. The composite reliability (CR) of the first dimension, Proactive Coping Strategies, was 0.891, with an average variance extracted (AVE) of 0.539. The second dimension, Engagement in the Learning Experience, had a composite reliability (CR) of 0.864 and an average variance extracted (AVE) of 0.679. The third dimension, Perceptions of Academic Adversity, had a composite reliability (CR) of 0.726 and an average variance extracted (AVE) of 0.475. According to Hair et al. (2017) in their work “A Primer on Partial Least Squares Structural Equation Modeling (PLS-SEM),” a CR greater than 0.7 is acceptable, even if the AVE is between 0.4 and 0.5. In summary, the CFA model in this study demonstrates good reliability, validity, and fit, as shown in Table 5.

**Table 5:** Summary table of factor analysis for each dimension.

Construct	Item	Explained variance	Cumulative explained variance	Factor loading	Cronbach’s α	CR	AVE
Proactive coping strategies	A7			0.764			
	A8			0.719			
	A6			0.696			
	A4			0.656			
	A15	42.02%	42.02%	0.649	0.899	0.891	0.539
	A16			0.604			
	A5			0.598			
	A11			0.564			
Engagement in the learning experience	A14			0.762			
	A13	14.7%	57.72%	0.738	0.871	0.864	0.679
	A12			0.715			
Perceptions of academic adversity	A1			0.786			
	A2	6.94%	63.66%	0.697	0.740	0.726	0.475*
	A3			0.618			

If the Composite Reliability (CR) is greater than 0.7, an Average Variance Extracted (AVE) between 0.4 and 0.5 can still be considered acceptable (Hair et al., 2017).

The analysis of the CFA scale’s dimensional structure resulted in a three-factor model named as follows: Proactive Coping Strategies: This factor, comprising items A4, A5, A6, A7, A8, A15, A16, and A11, reflects students’ proactive behaviors in handling academic setbacks. These behaviors include making coping plans, regulating emotions positively, maintaining a positive mindset, seeking alternative paths promptly, and sustaining enthusiasm for learning. Engagement in the Learning Experience: This factor, including items A12, A13, and A14, captures students’ positive emotional experiences during setbacks, such as enjoying learning and overcoming difficulties. Perceptions of Academic Adversity: This factor, comprising items A1, A2, and A3, describes students’ negative feelings during academic setbacks, like frustration and helplessness.

However, items A9, “My teachers always provide assistance when I encounter academic obstacles,” and A10, “My classmates encourage me not to give up on my studies,” were removed because their factor loadings in the CFA were insignificant for any single factor and correlated significantly with multiple factors. This indicates that teacher and peer support forms part of the external support system for university students, which is influenced by traditional Chinese cultural values like emotional restraint, stability, harmony, preserving “face,” and striving for improvement. These three factors comprehensively reflect students’ psychological states during academic setbacks, encompassing proactive coping, positive emotions, and negative feelings. This not only provides a theoretical basis for measuring students’ resilience but also informs educational practices to foster healthy student development. Detailed information on the Chinese University Students’ Academic Setback Resilience Scale (CUSASRS) is provided in Table 6.

**Table 6:** Chinese university students’ academic setback resilience (CUSASRS).

Construct of the theme	Construct item(s)
Perceptions of academic adversity	A1. The challenge I face is how to regain the optimal state of life and learning. A2. My challenge lies in how to actively face setbacks and reshape a positive self-image. A3. The challenge I face is how to regain the optimal state of life and learning.
Proactive coping strategies	A4. I will not give up my goals just because of temporary setbacks. A5. I am capable of overcoming academic obstacles. A6. Even when facing academic setbacks, I can quickly regain my determination A7. When confronted with setbacks, I weigh the pros and cons comprehensively to make the best choice. A8. I am confident that I will ultimately emerge from the shadows and completely overcome my current difficulties. A11. My family always supports my learning A15. I regulate my emotions through healthy and positive means to maintain a good mindset. A16. When facing developmental obstacles, I promptly seek favorable alternative paths for development.
Engagement in the learning experience	A12. No matter what kind of academic setbacks I experience, I can still enjoy the pleasure of learning. A13. The joy brought by learning often makes me forget the difficulties ahead
	A14. Even when encountering academic obstacles, I can continue to experience the joy of learning.

**Figure 2:**
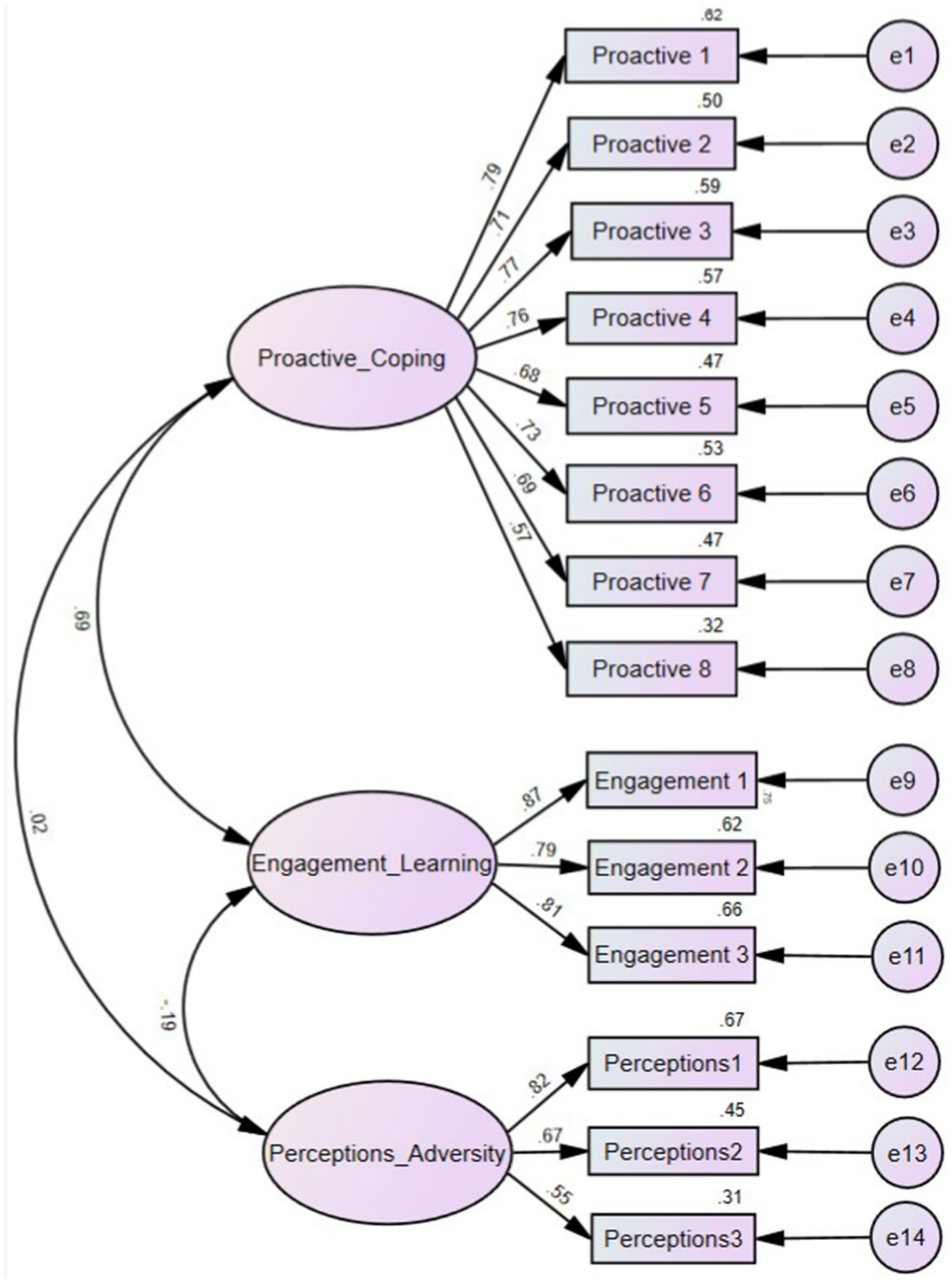
Confirmatory factor analysis (CFA).

Proactive Coping Strategies, Proactive1 (A8), Proactive2 (A7), Proactive3 (A6), Proactive 4 (A15), Proactive5 (A4), Proactive6 (A16), Proactive7 (A5), Proactive8 (A11). Engagement in the Learning Experience, Engagement 1 (A14), Engagement2 (A13), Engagement3 (A12). Perceptions of Academic Adversity, Perceptions1 (A2), Perceptions2 (A1), Perceptions3 (A3), Confirmatory factor analysis (CFA) is illustrated in Figure 2.

The closer the Goodness of Fit Index (GFI) is to 1, the higher the model fit. The GFI value of the model in this study is 0.927, and the Adjusted Goodness of Fit Index (AGFI) is 0.897. A GFI value greater than 0.9 indicates good model fit (Bentler, 1983; Hu and Bentler, 1999; Huang, 2007). An AGFI value greater than 0.8 also indicates good model fit (Doll et al., 1994). Therefore, the model in this study demonstrates a very good fit. The Root Mean Square Error of Approximation (RMSEA) value between 0.05 and 0.08 indicates a fair fit (McDonald and Ho, 2002; Huang, 2007). In this study, the RMSEA is 0.073, indicating a good model fit, as shown in Table 7.

**Table 7:** Analysis result of the overall model fit of the CUSASRS.

Goodness-of-fit indicators	Goodfit	Observed model	Appraisal
Absolute fit index			
Gfi	≧0.90	0.927	Good
Agfi	≧0.80	0.897	Good
Rmsea	≧0.05	0.073	Good
Nfi	≧0.90	0.918	Good
Incremental fit index			
IFI	≧0.90	0.939	Good
CFI	≧0.90	0.938	Good
TLI	≧0.90	0.924	Good
Parsimony fit index			
PGFI	≧0.50	0.654	Good
PNFI	≧0.50	0.747	Good
PCFI	≧0.50	0.763	Good
*x*^2^/df	≦3 or ≦5	3.776	Good

## 7 Discussion

### 7.1 Do qualitative interviews provide more accurate coding elements to reflect the academic setback experiences of Chinese university students?

Qualitative interviews offer a nuanced perspective on this matter. The final extracted three-factor structure of the scale deviates from the initial four constructs envisioned by the researchers, suggesting that theoretical constructs from Western backgrounds may not fully encompass the experiences of Chinese university students. Consequently, the scale was adjusted to include five constructs after integrating coding from qualitative research, which better captures students’ subjective experiences. Among the five constructs, a dimension labeled “external support” initially comprised three items. However, in factor analysis results, items referring to teachers and classmates were removed, suggesting that such support may not fully measure resilience to setbacks for Chinese students. Chinese students are less inclined to seek external resources from teachers or classmates, prioritizing independent problem-solving. This divergence from Western models reflects indigenous cultural characteristics of Chinese students in coping with setbacks. This also reflects the challenges encountered by Chinese university students during significant transitions, with family regarded as a supportive force. However, cultural traits influence how they cope with setbacks, making it difficult to seek help from sources beyond family due to concepts like “face” and the drive for perfection stemming from traditional educational ideals. Such nuanced descriptions provide deeper insights, often overlooked by Western scales, aiding in the refinement of scale items to better reflect indigenous cultural characteristics.

### 7.2 Has the integration of coding elements and items from Western scales resulted in a more effective and reliable localized academic setback resilience scale?

The statistical analysis of the final scale indicates significant reliability and validity, suggesting that integrating local coding with content from Western scales is feasible and effective. Initially comprising five constructs, including perceptions of academic adversity, internal positive forces, external support, positive emotional experiences, and proactive coping strategies, the questionnaire aimed to cover various aspects of academic setback resilience across cognitive, emotional, and behavioral domains.

However, confirmatory factor analysis (CFA) revealed only three main factors, resulting in a final scale of 14 items with a different item assignment. Proactive coping strategies incorporated items from the initial constructs of internal positive forces and proactive coping strategies, while the constructs of engagement in the learning experience and perceptions of academic adversity included all items from the initial scale. This suggests that, for Chinese university students, positive emotions and proactive behaviors are closely related when facing academic setbacks, forming an active engagement in learning. This integration better reflects the actual situation compared to distinct categorizations of emotions and behaviors. From a phenomenological perspective, this study thoroughly explores multiple dimensions of academic setback resilience, particularly perceptions of academic adversity. The construct of Perceptions of Academic Adversity reflects students’ internal psychological regulatory abilities, while Engagement in the Learning Experience emphasizes emotional factors. “Learning challenges” reflect objective difficulties, with students’ ability to cope primarily relying on intrinsic traits, contrasting Western scales’ emphasis on external support systems. Influenced by traditional Chinese cultural traits, Chinese students ideally utilize family support actively when facing setbacks, combining it with positive cognition, emotions, and behaviors. However, this also highlights the need for Chinese educational practices to integrate internal and external resources to promote students’ comprehensive development and enhance their overall resilience to setbacks.

## 8 Conclusion

The study aims to explore the genuine experiences of academic setbacks and coping strategies among Chinese university students using qualitative in-depth interviews. By coding interview content and integrating Western scales, a culturally appropriate assessment tool for academic setback resilience is developed, aiming to provide an effective measurement instrument for researching academic setback resilience among Chinese university students. Qualitative interviews capture the intricate emotional experiences of Chinese university students during academic setbacks, providing rich material for scale design. Participants’ descriptions of psychological processes during significant life transitions offer valuable insights into the subjective experiences of academic setbacks among Chinese university students. In Chinese culture, pursuing higher education is often linked to enhancing social status and supporting parents financially, contrasting with Western individualistic views. Thus, Chinese students’ attitudes toward setbacks differ, influenced by cultural traits like guilt, self-blame, risk aversion, reluctance to seek help to maintain “face,” and a drive for perfection rooted in traditional educational ideals. These cultural nuances shape the scale’s design, reflecting the subjective experiences of academic setbacks among Chinese university students. The development of the Chinese University Students’ Academic Setback Resilience (CUSASRS) scale provides a reliable tool for future cross-cultural research, aiding the study of academic setback resilience among Chinese individuals. In practical use, the scale can inform educational interventions to help university students enhance their coping abilities with academic setbacks.

This study makes several significant contributions:

Firstly, by conducting qualitative interviews, the study uncovers the subjective experiences and coping strategies of Chinese university students facing academic setbacks. This approach supplements quantitative questionnaire surveys and reveals unique responses and internal experiences, such as intense feelings of guilt, risk aversion, and concerns about seeking help due to cultural factors like “face” considerations. These findings deepen our understanding of academic setback resilience in Chinese culture and contribute empirical evidence for cross-cultural comparative studies. Secondly, the study sheds light on the complex emotional journeys of Chinese students during academic setbacks, providing insights into the need for internal processes of self-reflection and cognitive reshaping to regain positive emotions and academic motivation. This perspective informs the design of resilience measurement scales, offering a nuanced approach to assessing resilience in the face of setbacks. Moreover, by employing grounded theory to code interview data, the study enhances the cultural appropriateness of scale items and accurately reflects the unique psychological experiences of Chinese students. This methodological approach ensures the validity and reliability of the assessment tool. Lastly, the research findings provide a reliable assessment tool for psychological health education among Chinese university students, enabling schools to implement targeted intervention measures and promote students’ comprehensive development. Overall, this study contributes valuable insights into academic setback resilience in Chinese culture and informs practical interventions to support student well-being.

## Data Availability

The original contributions presented in the study are included in the article/Supplementary material, further inquiries can be directed to the corresponding author.
