# Supplementary material for: How can qualitative in-depth interviews optimize cross-cultural measurement of academic resilience?
Source: Front Psychol. 2025 Mar 20;16:1444978. doi: 10.3389/fpsyg.2025.1444978 (PMC11966960; doi:10.3389/fpsyg.2025.1444978)
Supplement: Supplementary file 1 [file Data_Sheet_1.pdf]

《大学生学业挫折容忍力相关研究》问卷

亲爱的同学：

您好！

我是岭南师范学院社会工作专业2021级的学生。感谢您拨冗参与本次问卷调查，为了更加详实地了解当代大学生学业挫折容忍力的实际状况，特邀您参加此次调查。本问卷共有两部分，请您认真填写基本资料，仔细阅读测量要求，按照自身实际情况进行填写。本次问卷调查采用匿名制，问卷数据仅供本人学术研究使用，绝不外泄，请放心填写。您的真实回答将对我们的研究提供宝贵的依据，谢谢您的配合，祝您生活愉快！

第一部分 基本资料

1.您的性别 [单选题]

○男

○女

2.您的年级 [单选题]

○大一

○大二

○大三

○大四

3.您的家庭所在地 [单选题]

○城市

○农村

4.您的学科 [单选题]

○文科

○理科

○音体美

○其他

5.是否独生 [单选题]

○是

〇否

第二部分 学业挫折容忍力量表

以下题目涉及个人感受，请您将它与您的真实情况进行比较，并在您认为合适的方框中打“√”。其中，1=非常不同意，2=不同意，3=有些不同意，4=不确定，5=有些同意，6=同意，7=非常同意。

		1	2	3	4	5	6	7
1	我的学习生活中经常会遇到逆境和困难							
2	学习任务太难使我感到沮丧							
3	我面临的难题是如何重拾生活和学习的最佳状态							
4	我不会因为暂时的失利就放弃目标							
5	我有能力应对遇到的学习障碍							
6	即使遇到学习挫折,我也能很快重拾斗志							
7	我会全面权衡利弊,做出最佳选择							
8	我有信心最终走出阴影,彻底克服当前的困境							
9	我的老师总能在我遇到学习障碍时给予帮助							
10	我的同学会鼓励我在学习上不放弃							
11	我的家人总是支持我学习							
12	不管经历了什么样的学习失利,我都能享受学习的乐趣							
13	学习带来的快乐常常使我忘记前途的困难							
14	即使遇到了学习障碍,我也能继续感受学习的乐趣							
15	我会通过健康正面的方式调节情绪,保持良好心态							

16	面临发展受阻,我会及时寻找有利的替代发展路径							

感谢您拨冗填写本问卷。您的意见对于我们的研究具有极大的学术价值，再次感谢您的宝贵时间和对我们研究的支持！
