# Supplementary material for: How can qualitative in-depth interviews optimize cross-cultural measurement of academic resilience?
Source: Front Psychol. 2025 Mar 20;16:1444978. doi: 10.3389/fpsyg.2025.1444978 (PMC11966960; doi:10.3389/fpsyg.2025.1444978)
Supplement: Supplementary file 2 [file Data_Sheet_2.pdf]

《The Chinese University Students' Academic Setback Resilience Scale》Questionnaire

Dear Student,

Greetings! I am a student of the 2021 cohort in the Social Work program at Lingnan Normal University. Thank you for taking the time to participate in this survey. To gain a more comprehensive understanding of the resilience to academic frustration among contemporary university students, you are invited to take part in this study. The questionnaire consists of two parts. Please complete the basic information section carefully, and read the measurement instructions thoroughly before answering based on your actual situation.

This survey is conducted anonymously, and the data collected will be used solely for academic research purposes. Rest assured that your responses will remain confidential. Your honest answers will provide invaluable insights for our research. Thank you for your cooperation, and we wish you a pleasant day!

Part I: Basic Information

Please indicate your gender: [Single Response]

○ Male

○ Female

What is your current year of study? [Single Response]

○ Freshman

○ Sophomore

○ Junior

○ Senior

Which best describes your hometown? [Single Response]

○ Urban area

○ Rural area

What is your primary field of study? [Single Response]

○ Humanities/Social Sciences

○ Natural Sciences

○ Visual/Performing Arts

○ Other (Please specify: ___________)

Are you an only child? [Single Response]

○ Yes

○ No

Part II: Academic Setback Resilience Scale

Please compare the following statements to your personal experiences and indicate your level of agreement by marking an "√" in the appropriate box. The scale is as follows: 1 = Strongly Disagree, 2 = Disagree, 3 = Somewhat Disagree, 4 = Neutral, 5 = Somewhat Agree, 6 = Agree, 7 = Strongly Agree.

		1	2	3	4	5	6	7
1	I often encounter adversity and difficulties in my academic life.							
2	Challenging academic tasks make me feel frustrated.							
3	My main challenge is how to regain the optimal state of living and learning.							
4	I do not abandon my goals due to temporary setbacks.							
5	I have the ability to overcome the learning obstacles I face.							
6	Even when faced with							

	academic setbacks, I can quickly regain my motivation.							
7	I thoroughly weigh the pros and cons to make the best choices.							
8	I am confident that I will eventually emerge from the shadows and completely overcome my current difficulties.							
9	My teachers always provide help when I encounter learning obstacles.							
10	My classmates encourage me not to give up on my studies.							
11	My family always supports my learning.							
12	No matter what kind of academic setbacks I experience, I can still enjoy the process of learning.							
13	The joy of learning often makes me forget about future difficulties.							
14	Even when encountering learning obstacles, I can continue to enjoy the							

	learning process.							
15	I regulate my emotions and maintain a positive mindset through healthy and constructive methods.							
16	When faced with developmental setbacks, I promptly seek alternative development paths.							

Thank you for taking the time to complete this questionnaire. Your feedback is of immense academic value to our research. We greatly appreciate your valuable time and support for our study!
